# Germ-Line Transmitted, Chromosomally Integrated HHV-6 and Classical Hodgkin Lymphoma

**DOI:** 10.1371/journal.pone.0112642

**Published:** 2014-11-10

**Authors:** Adam J. Bell, Alice Gallagher, Timothy Mottram, Annette Lake, Eleanor V. Kane, Tracy Lightfoot, Eve Roman, Ruth F. Jarrett

**Affiliations:** 1 MRC – University of Glasgow Centre for Virus Research, Glasgow, United Kingdom; 2 Epidemiology and Cancer Statistics Group, Department of Health Sciences, University of York, York, United Kingdom; IRCCS National Cancer Institute, Italy

## Abstract

A unique feature of both human herpesvirus 6A and B (HHV-6A and B) among human herpesviruses is their ability to integrate into chromosomal telomeres. In some individuals integrated viral genomes are present in the germ-line and result in the vertical transmission of HHV-6; however, little is known about the disease associations of germ-line transmitted, chromosomally integrated HHV-6 (ciHHV-6). Recent publications suggest that HHV-6 is associated with classical Hodgkin lymphoma (cHL). Here we examine the prevalence of ciHHV-6 in 936 cases of cHL and 563 controls by screening with a duplex TaqMan assay and confirming with droplet digital PCR. ciHHV-6 was detected in 10/563 (1.8%) controls and in all but one individual the virus was HHV-6B. Amongst cases 16/936 (1.7%) harboured ciHHV-6, thus demonstrating no association between ciHHV-6 and risk of cHL.

## Introduction

Recent publications suggest an association between human herpesvirus 6 (HHV-6) A and B and classical Hodgkin lymphoma (cHL) [1], [2]. cHL has a peak incidence in young adults (15–35 years) and epidemiological evidence suggests that an infectious agent may be involved in the aetiology of these cases [3]. A third of all cHL cases in industrialised countries are causally associated with Epstein-Barr virus (EBV); however, these cases are relatively more common in older adults and young children. It is therefore plausible that another virus is involved in the pathogenesis of EBV-negative cHL [3].

HHV-6A and -6B are two distinct but related human herpesviruses [4]. A feature unique to HHV-6A and B among human herpesviruses is their ability to integrate into the telomeres of human chromosomes [5], [6]. In a proportion of individuals the integrated virus is present in every nucleated cell in the body and passed to subsequent generations in a Mendelian fashion [7], [8]; this phenomenon is generally referred to as chromosomally integrated HHV-6 (ciHHV-6). The reported prevalence of ciHHV-6 among healthy individuals ranges from 0.8–4% [9].

Luppi *et al.* (1993) first described integrated HHV-6 genomes in three patients with high viral loads in peripheral blood; one of these patients had cHL and a second had a B-cell non-Hodgkin’s lymphoma [10]. Strenger *et al.* (2013) also documented ciHHV-6 in a cHL case, raising the possibility of a specific association between ciHHV-6 and cHL [2]. However, a recent study from Hubacek *et al.* (2013) did not find a difference in ciHHV-6 prevalence in patients with malignant disease and healthy controls [11]. Whilst this study did include 267 patients with cHL, it did not explicitly look at the prevalence of ciHHV-6 among these individuals. To conclusively determine whether ciHHV-6 is associated with cHL, we screened 936 cases of cHL and 563 healthy controls for ciHHV-6 using a quantitative PCR assay, and confirmed the presence of ciHHV-6 using droplet digital (ddPCR).

## Materials and Methods

### Subjects and Samples

Samples from 1508 subjects were analysed. These comprised: 375 cases and 349 controls from the Scotland and Newcastle Epidemiological Study of Hodgkin’s disease (SNEHD) [12]; 70 cases and 40 controls from the Young Adult Haematological malignancy and Hodgkin’s disease Case Control Study (YHHCCS) [13]; 170 cases and 174 controls from the Epidemiology and Genetics Lymphoma Case Control Study (ELCCS) [14]; and 321 cases from a prospective collection of newly diagnosed cHL cases in Scotland and the north of England. Subject characteristics including sex, age, tumour EBV status and histological subtype are summarised in Table 1. DNA was extracted from peripheral blood samples using standard procedures and subsequently whole genome amplified (WGA) (KBioscience, Herts, UK).

**Table 1:** Summary of patient characteristics for case and control groups of each sample set, and the whole study.

	SNEHD		YHHCCS		ELCCS		CaseSeries	Total	
	*N* (%)		*N* (%)		*N* (%)		*N* (%)	*N* (%)	
	Case	Control	Case	Control	Case	Control		Case	Control
**Total**	375(100)	349 (100)	70(100)	40(100)	170(100)	174(100)	321(100)	936(100)	563(100)
**Age Group (years)**									
<15	1(0.3)	0 (0)	0 (0)	0 (0)	0 (0)	0 (0)	11(3.4)	12(1.3)	0 (0)
15–34	185(49.3)	112 (32.1)	70 (100)	40(100)	70(41.2)	71(40.8)	159(49.5)	484(51.8)	223(39.6)
35–49	82(21.1)	92 (26.4)	0 (0)	0 (0)	53(31.2)	57(32.8)	69(21.5)	204(21.8)	149(26.4)
≥50	107(28.5)	145 (41.5)	0 (0)	0 (0)	47(27.6)	46(26.4)	82(25.6)	236(25.1)	191(34.0)
**Sex**									
Female	162(43.2)	148 (42.4)	33(47.1)	20(50)	66(38.8)	71(40.8)	144(44.9)	405(43.3)	239(42.4)
Male	213(56.8)	201 (57.6)	37(52.9)	20(50)	104(61.2)	103(59.2)	177(55.1)	531(56.7)	324(57.6)
**Histological Subtype**									
MC	80(21.3)	-	9(12.9)	-	29(17.1)	-	62(19.3)	180(19.2)	-
NS	257(68.6)	-	57(81.4)	-	133(78.2)	-	223(69.5)	670(71.6)	-
Other	38(10.1)	-	4(5.7)	-	8(4.7)	-	36(11.2)	86(9.2)	-
**EBV Status** *									-
Positive	98(30.0)	-	14(21.2)	-	33(31.1)	-	98(35.8)	243(31.4)	-
Negative	229(70.0)	-	52(78.8)	-	73(68.9)	-	176(64.2)	530(68.6)	-
Missing	48	-	4	-	64	-	47	163	-

*Percentage EBV-positive and negative only includes samples with known EBV status.

### Ethics Statement

Ethical approval was obtained from research ethics committees (REC) for each of the four contributing studies as follows: SNEHD, NHS Research Ethics Committee for Wales, REC reference number 08/MRE09/72; YHHCCS, NHS Greater Glasgow and Clyde West of Scotland Research Ethics Committee 4, REC reference number 09/S0704/73; ELCCS, NHS England Northern and Yorkshire Research Ethics Committee, REC reference number MREC/97/3/33; local case series, NHS Scotland Multi-Centre Research Ethics Committee for Scotland, REC reference number 06MRE00/83. All subjects provided written, informed consent.

### Quantitative PCR Assays

All samples were analysed using a duplex TaqMan qPCR assay that detects both the single copy *β-GLOBIN* gene and the *U7* gene of HHV-6A and B; both components of the assay have been described previously (Table 2) [15], [16]. Each 25 µl reaction contained 1×TaqMan Universal PCR MasterMix without UNG, *β-GLOBIN* primers at 50 nM and *U7* primers at 150 nM, both probes at 200 nM (all Life Technologies, Paisley, UK) and 50–100 ng of template DNA. Positive controls for the *U7* and *β-GLOBIN* assays consisted of the HHV-6A U7 amplicon region in pBlueScript (Dundee Cell Products, Dundee, UK) and HHV-6-negative human genomic DNA, respectively. Samples with known ciHHV-6 were also analysed in optimisation experiments. A no template control was included after every two samples. Amplification using default cycling parameters and results analysis were performed on a 7500 Fast Real-Time PCR system with Sequence Detection software v2.0.6 (Life Technologies). ΔC_T_ values (C_T_ *U7*– C_T_ *β-GLOBIN*) were used to determine whether a sample contained ciHHV-6. Where possible, cases suspected of having ciHHV-6 were retested using the original DNA sample, i.e. non-WGA DNA.

**Table 2:** Primers and probes used in screening assays, typing and ddPCR assays.

Name	Sequence
	(5′-3′)
*B-GLOBIN*Forward Primer	GGCAACCCTAAGGTGAAGGC
*B-GLOBIN*Reverse Primer	GGTGAGCCAGGCCATCACTA
*B-GLOBIN* Probe	6-FAM-ATGGCAAGAAAGTGCTCGGTGCCT-TAMRA
HHV-6 *U7*Forward Primer	AAAATTTCTCACGCCGGTATTC
HHV-6 *U7*Reverse Primer	CCTGCAGACCGTTCGTCAA
HHV-6 *U7* Probe	VIC-TCGGTCGACTGCCCGCTACCA-TAMRA
HHV-6A *POL*Forward Primer	GGCCAGCCAGTCCTTTAGTAGA
HHV-6A *POL*Reverse Primer	GGATGAGACTCATCGGTTTGTG
HHV-6A *POL* Probe	6-FAM-TCCCAAGCACAGACTCACGGATACAAGG-TAMRA*
HHV-6B *POL*Forward Primer	GGCCAGCCAGTCCTTTAGTAGA
HHV-6B *POL*Reverse Primer	GGATGAGACCCATCGGTTTGTG
HHV-6B *POL* Probe	6-FAM-TTCCAAGCACAGACTCGCGAACACAAGG-TAMRA*

*Reporter and quencher dyes were HEX and BHQ1, respectively for ddPCR.

Samples containing ciHHV-6 were further analysed to determine whether the virus was HHV-6A or B using previously described TaqMan qPCR assays detecting the *POL* gene of HHV-6A and HHV-6B (Table 2) [17]. qPCR was performed as above with the exception that primers were at 300 nM.

### Droplet Digital PCR

ddPCR copy number variant (CNV) analysis was used to confirm the presence of ciHHV-6 in positive samples identified in the qPCR screen. Duplex ddPCR incorporating a commercially available endogenous control assay (Human RPP30, Bio-Rad Laboratories, Hertfordshire, UK) and either the HHV-6A or HHV-6B *POL*, with the exception that probes were labelled with the reporter dye HEX and the dark quencher BHQ1 (Table 2).

To ensure even partitioning of DNA molecules into droplets, pre-digestion of the DNA is advised [18]. We utilised an ‘in-reaction’ digestion method, as described below, to avoid excessive manipulation of the sample before amplification.

ddPCRs were set up in duplicate in a 25 µl reaction volume. Each consisted of: 1×ddPCR Supermix for Probes without dUTP (Bio-Rad Laboratories); 1.25 µl of a 20×RPP30 primer/probe mix; viral specific primers and probes at 300 nM and 200 nM, respectively; 10–100 ng of template DNA and 1 µl of ‘digest mix’. The ‘digest mix’ consisted of 5 U µl^−1^ *Hind*III in 1x NEB buffer 2 (both New England BioLabs, Herts, UK). Twenty microlitres of the reaction were loaded into DG8 cartridges along with 70 µl of Droplet Generation Oil, and droplets were generated in a QX200 droplet generator (all Bio-Rad). The droplets were then transferred into 96-well PCR reaction plates (Eppendorf, Stevenage, UK). Thermal cycling was performed on a C1000 Touch Thermo Cycler (Bio-Rad) using the following conditions: 95°C for 10 minutes followed by 40 cycles of 94°C for 30 s and 60°C for 1 minute and a final extension at 98°C for 10 minutes. After amplification, results were analysed on a QX200 Droplet Reader using QuantaSoft analysis software v1.4.0. DNA samples extracted from ciHHV-6-positive lymphoblastoid cell lines (LCLs) were used as positive controls (kindly provided by Dr. D Clark, Royal Barts NHS Trust, London).

### Statistical Analysis

The proportion of individuals with ciHHV-6 in cases and controls, and subgroups stratified by age group and sex (all subjects), and histological subtype and EBV status (cases), were compared and Pearson’s chi-squared test was used to test whether differences were statistically significant. All statistical analyses were implemented using SPSS v19.0 (IBM, Middlesex, UK).

## Results

### ciHHV-6 screen of cHL cases and controls

Cases with ciHHV-6 generally contain one HHV-6 genome per diploid genome and therefore would be expected to contain a *β-GLOBIN* to *U7* copy number ratio of 2∶1. Using non-WGA DNA the components of the duplex TaqMan assay had similar efficiencies; however, *β-GLOBIN* standard curves, generated using ten-fold dilutions of PCR replicates, had a lower *y*-intercept than the corresponding *U7* standard curves resulting in a ΔC_T_ of approximately 3, rather than the expected ΔC_T_ of 1. Using WGA DNA, the *U7* component of the duplex TaqMan assay was less efficient than the *β-GLOBIN* component, and samples generally had higher C_T_ values in the *β-GLOBIN* assay, resulting in larger and more variable ΔC_T_ values All samples with a ΔC_T_≤6 were therefore considered likely to contain ciHHV-6. All samples had a *β-GLOBIN* C_T_≤32. Testing of replicates of WGA DNA from confirmed ciHHV-6-positive samples confirmed that ciHHV-6 was consistently detected when a C_T_ of 32 was observed in the *β-GLOBIN* assay.

Following the initial screen, 26 subjects were identified as potentially ciHHV-6-positive. The ΔC_T_ values of the respective samples ranged from 0.94–5.67. Non-WGA DNA was available from 21 of these subjects and analysis of these samples gave ΔC_T_ values in the range 2.61–3.69, with the exception of one sample (from subject 4298) which had a ΔC_T_ of 0.97 consistent with the presence of more than one integrated viral genome. Occasional other samples gave rise to low-level HHV-6 positive results but, of these, the lowest ΔC_T_ in the duplex assay was 10.4; thus samples with ciHHV-6 and exogenously acquired HHV-6 appeared clearly separable.

HHV-6 typing was performed on all 26 samples with ciHHV-6; two subjects were HHV-6A-positive and the remainder HHV-6B-positive.

### Confirmation of ciHHV-6 with ddPCR

To confirm the presence of ciHHV-6 in these samples we conducted ddPCR on the 21 samples with available material. ddPCR utilises reactions similar to TaqMan qPCR to quantify target sequences with a very high degree of accuracy. The QX200 ddPCR system (BioRad) partitions a 20 µl reaction volume into up to 20,000 droplets. PCR amplification occurs in each droplet and effectively increases the efficiency of the reaction by reducing competition between targets and confining the reaction components to a finite area. Each droplet acts as a single replicate and as the droplet volume is known this allows the absolute concentration of DNA target molecules to be calculated [18]. Recently ddPCR has been used as a means of detecting ciHHV-6 [19], [20]. We developed two duplex ddPCR assays that target the human *RPP30* gene and either the HHV-6A *POL* or HHV-6B *POL* gene; subjects were typed for HHV-6A and B, and the appropriate assay was used on each sample.

**Figure 1:**
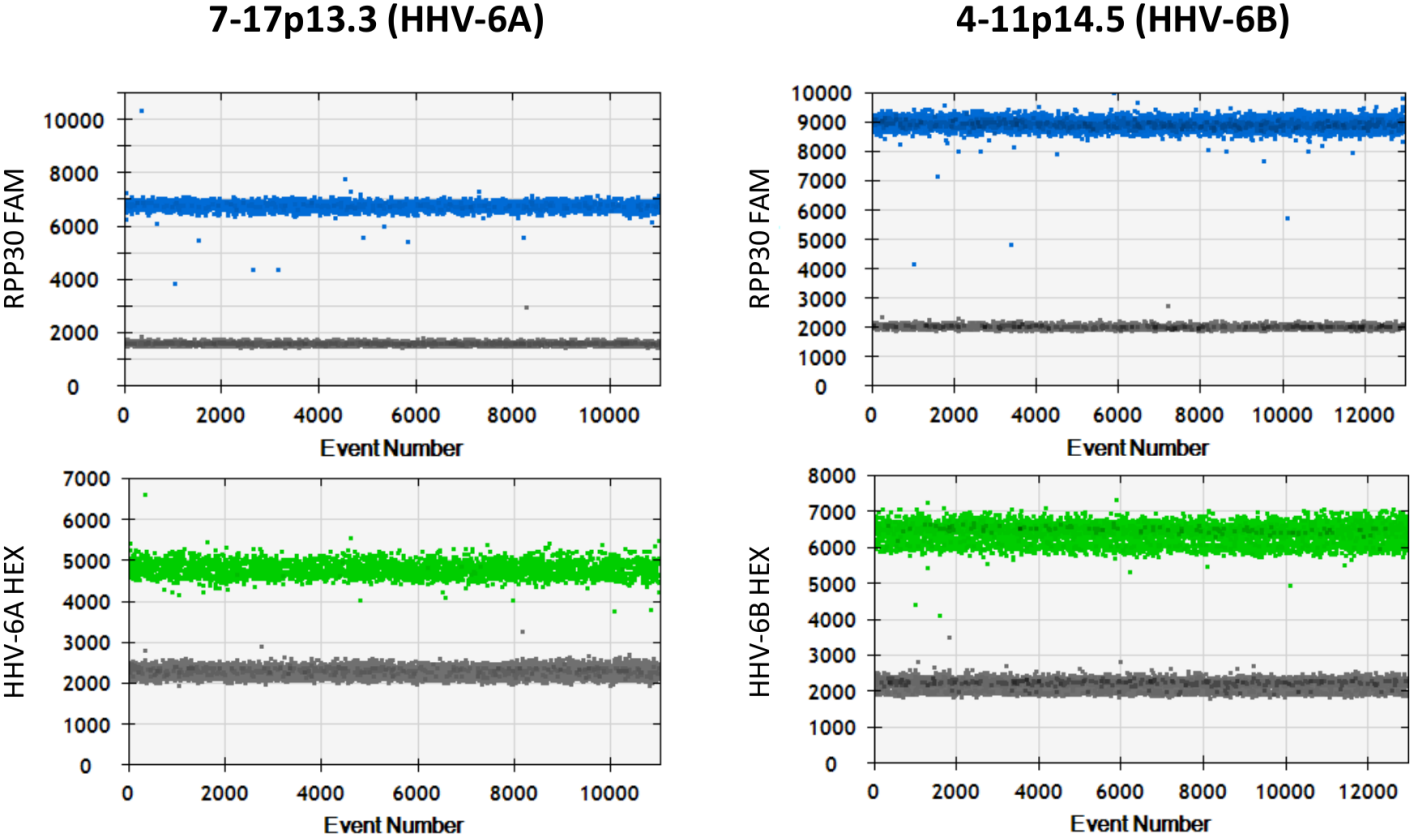
Analysis of ciHHV-6 by droplet digital PCR. One dimensional ddPCR plots for positive controls 7-17p13.3 (HHV-6A, panels A & B) and 4-11p14.5 (HHV-6B, panels C & D). Each control has two plots one representing the *RPP30* FAM (blue) channel (panels A & C) and the other the HHV-6 HEX (green) channel (panels B & D). Each point represents a single droplet, which is scored as positive (coloured) or negative (grey) depending on the fluorescent amplitude.

**Figure 2:**
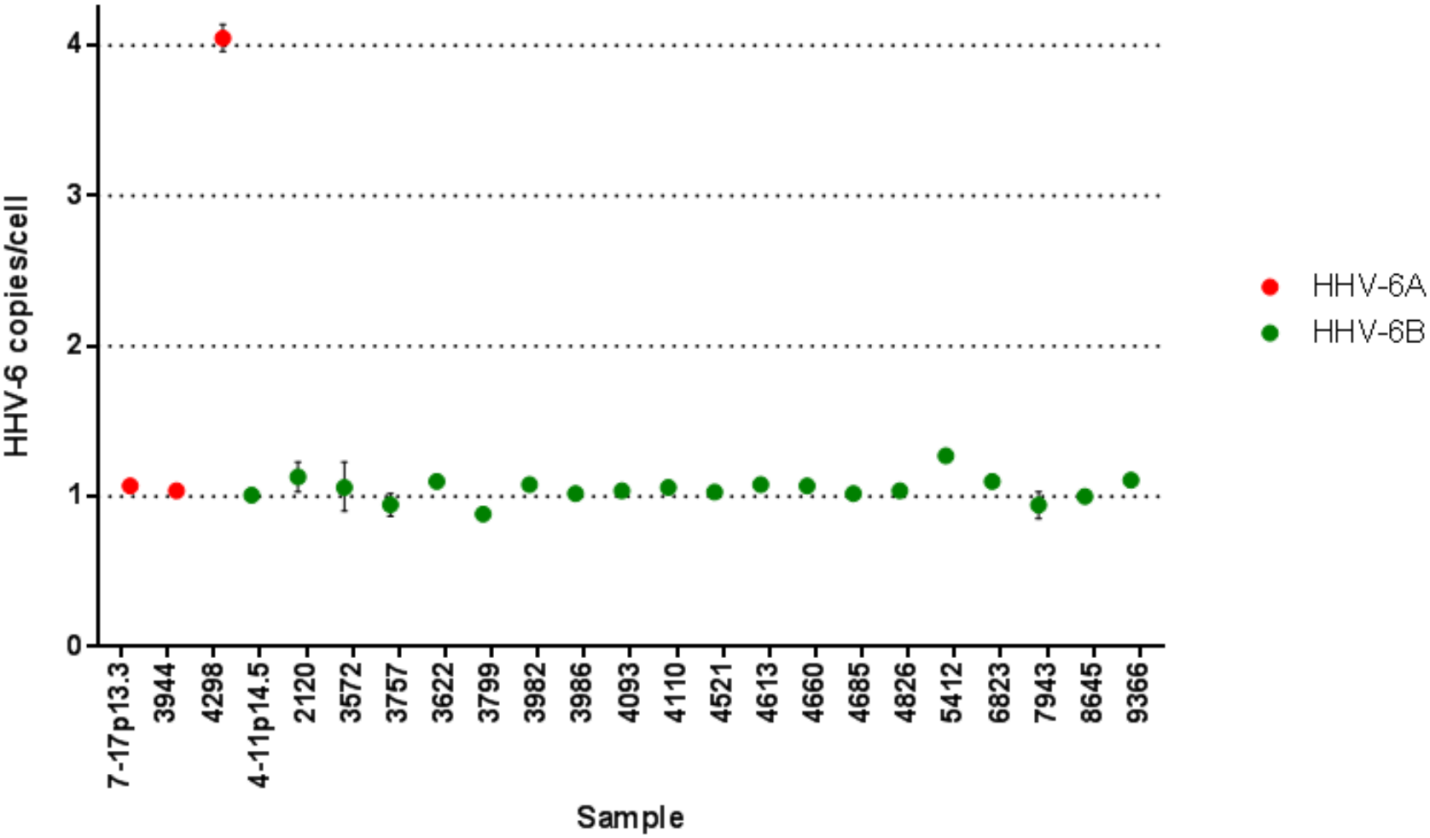
HHV-6 copies per cell in individuals identified as ciHHV-6-positive. Estimated HHV-6 genome copies per cell in subjects suspected to have ciHHV-6 and in positive controls (7-17p13.3 and 4-11p14.5). HHV-6A subjects are plotted in red and HHV-6B subjects in green. Error bars indicate the Poisson 95% confidence interval for each point.

The current gold standard for confirming ciHHV-6 is fluorescent *in* *situ* hybridisation (FISH); we therefore used DNA from LCLs, which had been confirmed to be ciHHV-6-positive by FISH, as positive controls. Both the HHV-6A and HHV-6B ddPCR duplex assays showed good separation of positive and negative droplets when run on ciHHV-6-positive LCL DNA (Fig. 1). The average number of HHV-6 genome copies per cell (gc/cell) was 1.07 and 1.01 for LCLs 7-17p13.3 (HHV-6A) and 4-11p14.5 (HHV-6A), respectively (Fig. 2). The appropriate duplex assay was conducted in duplicate on samples identified in the initial screen, with the exception of samples from 3572 and 3757 where DNA was limited. We analysed approximately 14,000 droplets per reaction. In all but one case the average HHV-6 copy number clustered around 1 gc/cell (range 0.884–1.2 gc/cell) (Fig. 2). The sample from subject 4298 had a HHV-6 copy number of 4.05 gc/cell (Poisson 95% confidence interval: 3.96, 4.14). ddPCR was repeated on this sample using a duplex assay which amplifies *RPP30* and the HHV-6 *U7* gene; the observed HHV-6 copy number was 3.95 gc/cell (Poisson 95% confidence interval: 3.69, 4.22). There was no evidence of off-target amplification, consistent with all these genomes being HHV-6A. These data suggest that subject 4298 has four integrated copies of HHV-6A per cell.

### ciHHV-6 prevalence in cHL cases and controls

Of the 26 ciHHV-6-positive subjects identified, 16 (1.7%) were cases and 10 (1.8%) controls; therefore, there was no statistically significant difference between cases and controls (*p* = 0.92). When cases and controls were broken down by study (Table 3 and Table S1) there was no evidence of heterogeneity. Of the two subjects with HHV-6A, one was a case and the other a control.

**Table 3:** Prevalence of ciHHV-6 in cases and controls by study.

			ciHHV-6 positive *N* (%)		
	Cases *(N)*	Controls *(N)*	Cases	Controls	*p* value
**SNEHD**	375	349	8 (2.1)	8 (2.3)	0.88
**YHHCCS**	70	40	1 (1.4)	0 (0)	-
**ELCCS**	170	174	2 (1.2)	2 (1.1)	0.98
**Case Series**	321	-	5 (1.6)	-	-
**Total**	**936**	**563**	**16 (1.7)**	**10 (1.8)**	**0.92**

The prevalence of ciHHV-6 was analysed by sex and age in four age groups: 0–14, 15–34, 35–49 and ≥50 years. No statistically significant differences were observed (data not shown). Cases were further analysed with respect to EBV status of tumours, histological subtype (nodular sclerosis versus mixed cellularity), and age in two groups (15–34 years and ≥50 years, to reflect the bimodal age distribution of cHL), or sex. No differences in prevalence were observed by EBV tumour status, histological subtype and sex. Older adult cases (7/236, 2.97%) were more likely to have ciHHV-6 than younger adult cases (5/484, 1.03%) and this difference approached statistical significance (*p* = 0.057). The difference between these two age groups among controls (4/223, 1.79% and 4/191, 2.09%, respectively) was not significant (*p* = 0.83).

## Discussion

This is the largest study, to date, to investigate the prevalence of ciHHV-6 among cHL patients, and also one of the largest screens for ciHHV-6 in control subjects in the UK. We identified 26 subjects including 16 (1.7%) cHL cases and 10 (1.8%) controls with ciHHV-6. There was no difference in the prevalence of ciHHV-6 in cHL cases and controls, suggesting that inheritance of an integrated HHV-6 genome does not increase risk of this disease. The prevalence of ciHHV-6 among control subjects reported here is within the range previously reported for Western populations and the proportion of positive subjects is not significantly different from other studies of controls with sample size ≥100 [9], [11], [21]–[24].

Further analysis of cases did not reveal any association between prevalence of ciHHV-6 and EBV status or histological subtype of cHL. When cases were stratified by age group, older adult cases were found to be more frequently ciHHV-6-positive than younger adult cases and these differences approached statistical significance (*p* = 0.057). Given the small number of ciHHV-6-positive samples and lack of case control differences, this is likely to be due to chance; however, since HHV-6 integrates into telomeres and telomeres are involved in cell senescence [25], it remains possible that this is biologically relevant and therefore this should be addressed in future studies.

The results confirm that ddPCR is more precise than conventional TaqMan PCR for detecting the presence of ciHHV-6 in DNA samples. The HHV-6 genome copy number estimates clustered around values which suggest inherited genomes. We observed a copy number range of 0.884–1.2 gc/cell in all individuals except subject 4298, and in most cases the average value was extremely close to 1 with 95% confidence intervals approaching or crossing 1. Failure to detect a copy number of exactly 1 may relate to the age of the samples as recent reports in the literature suggest that sample age can affect CNV analysis results [26]. The ciHHV-6-positive samples analysed in this study were collected between 1992 and 2009 and therefore sample DNA was between 5 and 22 years old. However, it cannot be ruled out that subjects with a HHV-6 copy number >1 gc/cell have some degree of active infection, and that subjects with a HHV-6 copy number <1 gc/cell have some cells that have deleted all or part of their viral genomes, as has been described recently [23].

We identified and report for the first time an individual who is likely to have four integrated copies of HHV-6A. There has been a reported case of ciHHV-6 with two integrated viruses; in this case it was confirmed that a single copy HHV-6 genome had been inherited from each parent [8]. It is possible that this individual has inherited two integrated viruses from each parent; however, other possibilities such as new primary integration events, reactivation and further integration of already present virus or integration of viral genome concatemers cannot be excluded.

Exogenously acquired HHV-6A appears to have a low prevalence in industrialised countries. In the USA, 97–100% of all symptomatic and asymptomatic primary infections in infants are HHV-6B [27]–[29]. This is mirrored in European populations where up to 95% of primary infections are HHV-6B [30], [31]. In contrast, in Sub-Saharan Africa, HHV-6A is the dominant virus accounting for 86% of asymptomatic infections in Zambian infants [32]. It has been suggested previously that between one-fifth and one-third of ciHHV-6 genomes are HHV-6A [33]. Given that most of the contributing studies analysed individuals from industrialised countries, this in turn leads to the suggestion that HHV-6A may integrate more frequently than HHV-6B. In studies with ten or more ciHHV-6-positive subjects HHV-6A has accounted for 6.8–40% of ciHHV-6 genomes [22], [23], [34]. In the present study we identified only 2 subjects (8%) with ciHHV-6A. These data are more consistent with the idea that the proportion of HHV-6A among ciHHV-6-positive subjects reflects the proportions of HHV-6A and HHV-6B in the underlying population. Both ciHHV-6A subjects identified in this study are white British.

In conclusion, the data presented here suggest that the prevalence of ciHHV-6 in the UK is ∼1.8% but do not support an association between ciHHV-6 and cHL risk.

## Supporting Information

Table S1 **Further characteristics of subjects included in this study.**
(XLSX)
